# Molecular evidence suggesting the persistence of residual SARS‐CoV‐2 and immune responses in the placentas of pregnant patients recovered from COVID‐19

**DOI:** 10.1111/cpr.13091

**Published:** 2021-07-22

**Authors:** Hao Wu, Shujie Liao, Yiming Wang, Ming Guo, Xingguang Lin, Jianli Wu, Renjie Wang, Dan Lv, Di Wu, Mengzhou He, Bai Hu, Rui Long, Jing Peng, Hui Yang, Heng Yin, Xin Wang, Zhixiang Huang, Ke Lan, Yanbin Zhou, Wei Zhang, Zhenyu Xiao, Yun Zhao, Dongrui Deng, Hongmei Wang

**Affiliations:** ^1^ State Key Laboratory of Stem Cell and Reproductive Biology Institute of Zoology Chinese Academy of Sciences Beijing China; ^2^ Institute for Stem Cell and Regeneration Chinese Academy of Sciences Beijing China; ^3^ University of Chinese Academy of Sciences Beijing China; ^4^ Beijing Institute for Stem Cell and Regenerative Medicine Beijing China; ^5^ Department of Gynecology and Obstetrics Tongji Hospital Tongji Medical College Huazhong University of Science and Technology Wuhan China; ^6^ State Key Laboratory of Virology Modern Virology Research Center College of Life Sciences Wuhan University Wuhan China; ^7^ Department of Obstetrics, Maternal and Child Health Hospital of Hubei Province Tongji Medical College Huazhong University of Science and Technology Wuhan China; ^8^ Department of Obstetrics and Gynecology People’s Hospital of Huangmei Country Huanggang City China; ^9^ Zhongnan Hospital Wuhan University Wuhan China

**Keywords:** COVID‐19, cytokine, immune response, placenta, pregnant women, SARS‐CoV‐2

## Abstract

**Objectives:**

Recent studies have shown the presence of SARS‐CoV‐2 in the tissues of clinically recovered patients and persistent immune symptoms in discharged patients for up to several months. Pregnant patients were shown to be a high‐risk group for COVID‐19. Based on these findings, we assessed SARS‐CoV‐2 nucleic acid and protein retention in the placentas of pregnant women who had fully recovered from COVID‐19 and cytokine fluctuations in maternal and foetal tissues.

**Materials and Methods:**

Remnant SARS‐CoV‐2 in the term placenta was detected using nucleic acid amplification and immunohistochemical staining of the SARS‐CoV‐2 protein. The infiltration of CD14+ macrophages into the placental villi was detected by immunostaining. The cytokines in the placenta, maternal plasma, neonatal umbilical cord, cord blood and amniotic fluid specimens at delivery were profiled using the Luminex assay.

**Results:**

Residual SARS‐CoV‐2 nucleic acid and protein were detected in the term placentas of recovered pregnant women. The infiltration of CD14+ macrophages into the placental villi of the recovered pregnant women was higher than that in the controls. Furthermore, the cytokine levels in the placenta, maternal plasma, neonatal umbilical cord, cord blood and amniotic fluid specimens fluctuated significantly.

**Conclusions:**

Our study showed that SARS‐CoV‐2 nucleic acid (in one patient) and protein (in five patients) were present in the placentas of clinically recovered pregnant patients for more than 3 months after diagnosis. The immune responses induced by the virus may lead to prolonged and persistent symptoms in the maternal plasma, placenta, umbilical cord, cord blood and amniotic fluid.

## Graphical abstract

Remnant SARS‐CoV‐2 virus RNA and protein could be detected in the term placentas of recovered pregnant women. Immune responses of maternal plasma, placenta, umbilical cord, cord blood, and amniotic fluid were activated by the viruses may persist even longer *in vivo*

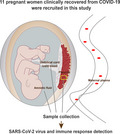

## 1 INTRODUCTION

As of April 19, 2021, more than 146 million individuals have been diagnosed with coronavirus disease (COVID‐19) caused by severe acute respiratory syndrome coronavirus 2 (SARS‐CoV‐2) (Data from WHO). The major manifestations of COVID‐19 are observed in the respiratory organs.^1^, ^2^, ^3^, ^4^ Patients may also show gastrointestinal and neurological symptoms and myocardial dysfunction.^5^, ^6^, ^7^, ^8^, ^9^ Most patients develop unilateral or bilateral pneumonia, which is diagnosed by radiological examination.^10^ In patients with severe COVID‐19, the disease can rapidly progress into acute respiratory distress syndrome (ARDS), severe sepsis with shock or multiple organ failure within 1 week due to the onset of a ‘cytokine storm’.^11^

SARS‐CoV‐2 is primarily transmitted through the respiratory tract and infects airway epithelial cells, vascular endothelial cells and macrophages.^12^, ^13^ The cellular entry of SARS‐CoV‐2 is mediated by the spike (S) protein. The binding of the S protein to the cell surface receptor angiotensin‐converting enzyme 2 (ACE2) exposes a cleavage site on the S protein. Transmembrane protease serine 2 recognizes this cleavage site and proteolytically cleaves the S protein to initiate fusion and endocytosis.^14^, ^15^ Cells in various human tissues, including the small intestine, testes, kidney, heart, thyroid, adipose and placenta, show high *ACE2* expression, whereas the lung cells show moderate expression.^16^, ^17^ Consistently, autopsies in cases of severe COVID‐19 have shown that in addition to the lungs, the virus infects various tissues, including the heart, kidneys and liver, among others.^4^ These findings provide evidence of the systemic spread of SARS‐CoV‐2 in the body during infection.

Following infection, an immune response is typically induced against the pathogen, and patients with severe COVID‐19 may suffer from lymphocytopenia and macrophage activation syndrome.^18^, ^19^ Moreover, there are reports of increased secretion of a series of cytokines and chemokines in the plasma, including interleukin (IL)‐2, IL‐7, IL‐10, granulocyte colony‐stimulating factor, interferon (IFN)‐gamma‐induced protein 10 (IP‐10), monocyte chemoattractant protein‐1 (MCP‐1), macrophage inflammatory protein 1 alpha and tumour necrosis factor‐alpha (TNF‐α). The plasma cytokine profile of patients was shown to be associated with COVID‐19 severity.^20^, ^21^, ^22^

Pregnant women are a high‐risk population for severe COVID‐19 and exhibit high mortality owing to their unique immune status.^21^, ^23^ Maternal inflammatory responses at the maternal–foetal interface, mediated through macrophages and T cells, are induced after SARS‐CoV‐2 infection, and these responses could persist for as long as 3 months after COVID‐19 recovery.^4^, ^24^, ^25^, ^26^

During pregnancy, the placenta acts as a transient endocrine organ that supports foetal growth by extracting nutrients from the maternal blood and serving as a barrier against pathogens or mediators of the maternal immune system. The placental villus is a functional unit of the placenta, composed of a layer of mononucleated cytotrophoblast cells and an outer multinucleated syncytiotrophoblast.^27^ Within the placental villi, Hofbauer cells (placental macrophages), placental fibroblasts and foetal endothelial cells are located adjacent to the foetal capillaries. The presence of Hofbauer cells is important for a successful pregnancy, as it regulates placental morphogenesis and the immune system.^28^ Although placental SARS‐CoV‐2 infection may damage the syncytiotrophoblast and disrupt the placental barrier,^29^ the presence of SARS‐CoV‐2 in the placenta post‐recovery and the chances of its vertical transmission are debatable, and the impact of SARS‐CoV‐2 on the placenta and foetus should be investigated further.

In this study, we used the placentas, maternal plasma, neonatal umbilical cords, cord blood and amniotic fluid donated at delivery by pregnant patients who had completely recovered from COVID‐19 and confirmed the presence of residual SARS‐CoV‐2 nucleic acid and protein in the placentas of patients with COVID‐19 long after initial diagnosis and complete recovery. The longest interval between diagnosis and sampling was approximately 3 months, and we believe that the immune response induced by SARS‐CoV‐2 may persist even longer.

## 2 MATERIALS AND METHODS

### 2.1 Patients

The biological specimens used in this study were collected from 11 pregnant women who had recovered from COVID‐19 in Wuhan, China, between March 30 and April 24, 2020. The women were diagnosed with COVID‐19 by the local Center for Disease Control or by a designated diagnostic laboratory in accordance with the novel coronavirus pneumonia diagnosis and treatment program (5th edition). Among the women, four (#30, #32, #47 and #49) had an asymptomatic infections. At the time of delivery, the patients met the requirements for clinical discharge or release from quarantine, with approval provided by clinicians. However, owing to the limited information about the pathogen and the lack of sensitive testing methods at the onset of suspected symptoms, the time point of initial infection could not be determined in some patients. All the patients were treated in isolation. The diagnosis was based on SARS‐CoV‐2 nucleic acid detection from throat swabs, serum antibody testing and chest CT imaging before delivery (Table S2). Four patients (#14, #20, #46 and #49) underwent RNA and antibody testing after delivery. Maternal plasma, placentas, neonatal umbilical cords, cord blood and amniotic fluid were collected under stringent conditions to eliminate the chances of accidental contamination, and all samples were stored in a biosafety level 3 containment laboratory. The experiments were performed after temperature inactivation, with liquid samples processed at 56 ºC for 45 min and solid samples processed at 95 ºC for 45 min. All researchers had requisite qualifications for biosafety and experimental operations. The experimental procedures complied with the standard instructions and requirements of the Institute of Zoology, Chinese Academy of Sciences.

### 2.2 Ethical considerations

The study was approved by the Research Ethics Committee (reference number: TJ‐IRB20200732) of Tongji Hospital, Tongji Medical College, Huazhong University of Science and Technology. All participants (patients and uninfected pregnant women) who donated biological specimens provided written informed consent.

### 2.3 Real‐time fluorescent quantitative polymerase chain reaction (qRT‐PCR)

RNA extracted from maternal throat swabs, placental villi (10 mg) and neonatal throat swabs were used to test for SARS‐CoV‐2 S protein using qRT‐PCR. The RNA titre was measured using a fluorescent probe targeting the S protein RNA. The sequences of the probes were as follows: CoV‐F (5’‐TCCTGGTGATTCTTCTTCAGGT‐3’), CoV‐R (5’‐TCTGAGAGAGGGTCAAGTGC‐3’) and CoV‐P (5’‐FAM‐AGCTGCAGCACCAGCTGTCCA‐BHQ1‐3’). Samples were considered SARS‐CoV‐2‐positive when the cycle threshold values for S protein were ≤ 38.^30^ Viral nucleic acid detection was performed for six placental villi specimens (#26, #30, #42, #46, #47 and #49). RNA extracted from the term placental villi of three uninfected pregnant women was used as the negative control.

### 2.4 Anti‐SARS‐CoV‐2 IgG and IgM testing

ELISA was used to detect SARS‐CoV‐2‐specific IgG and IgM in maternal serum, neonatal serum, umbilical cord, cord blood and amniotic fluid specimens, as instructed.^31^ In total, maternal plasma (collected at delivery) from 11 patients, neonatal serum from seven patients (#26, #27, #32, #35, #46, #47 and #49), umbilical cords from five patients (#26, #27, #46, #47 and #49) and amniotic fluid from three patients (#26, #42 and #47) were tested for SARS‐CoV‐2‐specific antibodies.

### 2.5 Immunohistochemical staining

Immunohistochemical staining was performed as described by Fu et al^32^ Briefly, 5‐μm‐thick paraffin sections were treated with mouse monoclonal anti‐SARS‐CoV‐2‐S (Sino Biological, 40150‐T62‐COV2, 1:200),^33^, ^34^ mouse monoclonal anti‐β‐hCG (ZSGB‐BIO, ZM‐0134, 1:200), rabbit polyclonal anti‐CD14 (ProteinTech, 17000‐1‐AP, 1:400) and rabbit monoclonal anti‐CD3e (Invitrogen, MA5‐14524, 1:200) antibodies. After washing with phosphate‐buffered saline (PBS) thrice, the slides were treated with anti‐mouse or anti‐rabbit secondary antibodies using a two‐step immunohistochemistry kit (ZSGB‐BIO, PV‐9001; ZSGB‐BIO, PV‐9002). Positive signals were indicated by brown staining with diaminobenzidine, and the nuclei were stained blue with haematoxylin. Term placentas from three uninfected pregnant women were used as negative control specimens. The immunostained specimens were imaged using a microscope (ZEISS, AX10). The percentage of positive nuclei among the total nuclei was calculated using Image Processing and Analysis in Java (ImageJ).

### 2.6 Cytokine and chemokine measurement

To characterize the cytokine profiles of maternal plasma, placenta, neonatal umbilical cord, cord blood and amniotic fluid specimens, the Luminex assay was performed using the Bio‐Plex Pro Human Cytokine Screening Panel (Bio‐Rad Laboratories, Hercules, CA, USA), which can detect 48 cytokines and chemokines. The data were collected on a Luminex 200 Instrument System and analysed using Luminex xPONENT (Thermo Fisher Scientific, Waltham, MA, USA). The mean cytokine level in uninfected controls was considered the baseline and the cytokine levels in the specimens were expressed relative to the baseline level and termed "relative secretion levels". For negative controls, we used maternal plasma, placenta, umbilical cord, cord blood and amniotic fluid donated by three, four, two, two and three uninfected pregnant women at delivery respectively.

### 2.7 Statistical analysis

Results are expressed in terms of means ± standard error of the mean. Statistical analysis was performed using a paired‐sample t‐test with Statistical Package for Social Science (SPSS; SPSS Inc., Chicago, IL, USA). Significance was defined as follows: *, *P* <.05; **, *P* <.01; ***, *P* <.001.

## 3 RESULTS

### 3.1 Clinical manifestations

The clinical details of the pregnant patients with COVID‐19 and their fetuses have been summarized in Tables 1 and 2. The pregnant women had been discharged from the hospital before delivery, and routine prenatal examinations were performed after the completion of the quarantine period (Tables S1 and S2). After recovery, the women delivered 11 infants (one set of monochorionic diamniotic twins). Five patients underwent caesarean deliveries, five patients had natural childbirth and one patient experienced foetal malformation‐induced labour. The twins were delivered preterm at 36^+4^W, whereas the other infants were full‐term newborns (Table S3). Subsequent assessments were based on the biological samples obtained.

**TABLE 1:** Clinical manifestations of the recovered pregnant women from COVID‐19

	**#14**	**#20**	**#26**	**#27**	**#30**	**#32**	**#35**	**#42**	**#46**	**#47**	**#49**
**Demographics**											
Age (y)	29	25	29	33	32	26	31	33	34	34	30
Gestational age of first onset of symptoms	N/A	N/A	31^+2^W	28^+5^W	Asymptomatic	Asymptomatic	27^+5^W	N/A	22^+6^W	Asymptomatic	Asymptomatic
**Comorbidities**											
Gestational hypertension	No	Yes	No	No	No	No	No	No	No	No	Yes
ICP	No	No	No	No	No	No	No	No	No	No	No
Hepatitis B	No	No	No	No	No	No	No	No	No	No	No
GDM	No	No	No	No	No	No	No	Yes	Yes	No	No
Other **╛**	No	No	No	No	No	No	No	No	No	No	No
**Diagnosis criteria for admission ╧**											
Nucleic acid of SARS‐CoV‐2 in throat swab	Negative	Negative	Positive	Negative	Negative	Negative	Positive	Negative	Positive	Negative	Negative
Anti‐SARS‐CoV‐2 antibodies in serum	IgG+/IgM‐	IgG+/IgM‐	IgG+/IgM‐	IgG+/IgM‐	IgG+/IgM‐	IgG+/IgM‐	IgG+/IgM‐	IgG+/IgM‐	IgG+/IgM‐	IgG+/IgM‐	IgG+/IgM‐
Clinically‐determined	Yes	Yes	No	Yes	Yes	Yes	No	Yes	No	Yes	Yes
**Signs and symptoms on admission**											
Asymptomatic	No	No	No	No	Yes	Yes	No	No	No	Yes	Yes
Fever on admission	No	No	Yes	Yes	No	No	Yes	Yes	Yes	No	No
Other signs and symptoms	Cough	Cough	No	Cough	No	No	Cough	Cough	Cough	No	No
**First radiologic examination (Chest CT)**											
Abnormalities on chest CT	Yes	Yes	Yes	Yes	Yes	No	Yes	No	No	No	No
Ground‐glass opacity	No	No	Yes	Yes	No	No	No	No	No	No	No
Local patchy shadowing	Yes	Yes	Yes	No	No	No	No	No	No	No	No
Others	No	No	No	No	Nodule	No	Bullae, Fibrosis	No	No	No	No
**Degree of severity ╩**	Moderate	Moderate	Moderate	Moderate	Moderate	Mild	Moderate	Mild	Mild	Mild	Mild
**Outcomes**	Discharge	Discharge	Discharge	Discharge	Discharge	Discharge	Discharge	Discharge	Discharge	Discharge	Discharge

Abbreviations: ╛, Other complications include respiratory failure, heart failure, kidney failure, sepsis and shock; ╧, On admission, the patients were confirmed by positive viral nucleic acid in throat swab or according to typical clinical symptoms and chest CT imaging; ╩, Refer to National Health Commission of the People's Republic of China novel coronavirus pneumonia diagnosis and treatment program (5th edition) (in Chinese); GDM, Gestational diabetes mellitus; ICP, Intrahepatic cholestasis of pregnancy; N/A, Not applicable; W, Week; y, Year old.

**TABLE 2:** SARS‐CoV‐2 detection in the newborns

	**#14**	**#20**	**#26**	**#27**	**#30**	**#32**	**#35**	**#42**	**#46**	**#47**	**#49**
**Nucleic acid of SARS‐CoV‐2 detections**											
Throat swab	N/A	Negative	Negative	Negative	N/A	N/A	Negative	N/A	N/A	N/A	N/A
**Anti‐SARS‐CoV‐2 antibody detections**											
Serum	N/A	N/A	IgG+/IgM‐	IgG+/IgM‐	N/A	IgG+/IgM‐	IgG+/IgM‐	N/A	IgG+/IgM‐	IgG+/IgM‐	IgG+/IgM‐
Umbilical cord	N/A	N/A	IgG+/IgM‐	IgG+/IgM‐	N/A	N/A	N/A	N/A	IgG+/IgM‐	IgG+/IgM‐	IgG+/IgM‐
Amniotic fluid	N/A	N/A	IgG+/IgM‐	N/A	N/A	N/A	N/A	IgG‐/IgM‐	N/A	IgG+/IgM‐	N/A

Abbreviation: N/A, Not applicable.

### 3.2 Laboratory detection of residual SARS‐CoV‐2 nucleic acid and protein in the placenta

**FIGURE 1:**
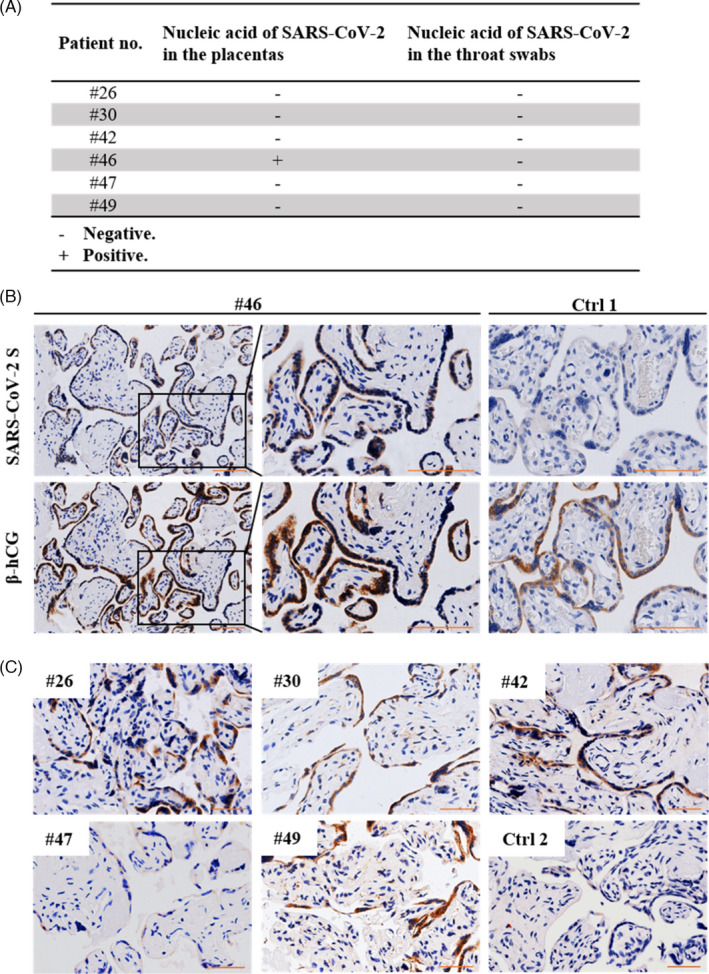
Detection of SARS‐CoV‐2 nucleic acid in term placentas and throat swabs. A, Results of SARS‐CoV‐2 qRT‐PCR showing the expression of viral S gene RNA in term placentas and maternal throat swabs collected at delivery. The samples were considered to test positive at cycle threshold (Ct) values ≤38. B, Immunostaining for viral S protein and β‐hCG (a syncytiotrophoblast (STB) marker) on consecutive sections of term placental villi from patient #46. Term placental villi from an uninfected pregnant woman served as the negative control (Ctrl 1). Scale bars: 100 μm. C, Immunostaining for viral S protein in the placentas of patients #26, #30, #42, #47 and #49. The placenta of an uninfected pregnant woman served as the negative control (Ctrl 2). Scale bars: 50 μm

To explore the existence of SARS‐CoV‐2 in the placenta of clinically recovered patients, we first performed qRT‐PCR on isolated total RNAs of the placentas from six patients (#26, #30, #42, #46, #47 and #49). As shown in Figure 1A, the placenta from patient #46 tested positive for viral nucleic acid, whereas the other placentas tested negative. To determine whether the viral protein was present in the placenta, we performed immunohistochemical staining for the SARS‐CoV‐2 spike (S) protein of the six placentas (#26, #30, #42, #46, #47 and #49). S protein was detected at high levels in the placental villi of five patients (#26, #30, #42, #46 and #49) (Figure 1B and 1C). The immunostaining pattern of β‐hCG, a syncytiotrophoblast marker, in serial sections, indicated that the S protein was primarily localized in the syncytiotrophoblast rather than in cytotrophoblast cells or other placental cells. The specificity of the antiviral antibody was determined by immunostaining the sections directly with a second antibody, which did not yield positive signals (Figure S1). Interestingly, even though the placentas from patients #30 and #49 showed the presence of SARS‐CoV‐2 protein, the patients had asymptomatic infection (Table 1), suggesting that the viral protein may be retained in the placenta of pregnant patients with asymptomatic infection who tested negative for SARS‐CoV‐2 and were released from medical quarantine. The presence of both SARS‐CoV‐2 nucleic acid and protein in the placenta indicated that the placenta is vulnerable to SARS‐CoV‐2 infection and the virus persists in the placenta long after the patients are discharged from the hospital.

**FIGURE 2:**
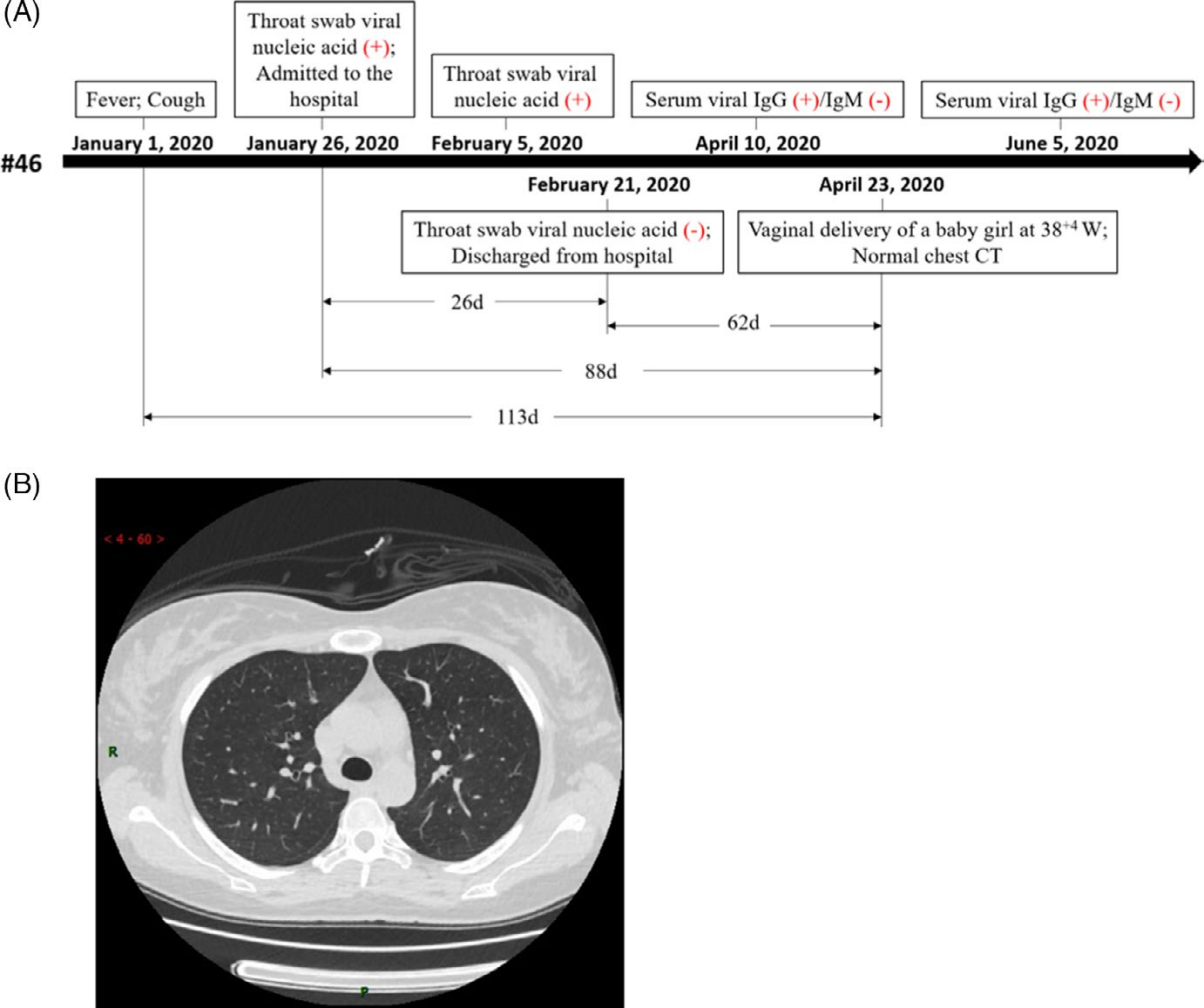
COVID‐19 clinical testing of patient #46. A, Timeline of COVID‐19 testing of patient #46 starting with the suspected symptom onset. B, Chest CT image showing the normal condition of the lungs of patient #46 before delivery

To estimate the duration of SARS‐CoV‐2 retention in the placenta, we analysed the clinical course of patient #46, who showed the presence of both SARS‐CoV‐2 nucleic acid and protein in the placenta. As shown in Figure 2A, in the case of patient #46, a 34‐year‐old pregnant woman, there was an interval of 88 days between COVID‐19 diagnosis and sampling. The patient experienced fever and cough on January 1, and the throat swab samples tested positive for the SARS‐CoV‐2 S gene, as examined on January 26 and February 5. The patient was admitted to the hospital on January 26. The throat swab sample collected on February 21 tested negative for SARS‐CoV‐2, following which the patient was discharged. On April 10, the plasma sample collected from the patient showed the presence of anti‐SARS‐CoV‐2 IgG, whereas IgM was not detected. The patient experienced labour onset on April 23, and a normal chest CT report was obtained at delivery (Figure 2B). There was a gap of 113 days between the suspected onset of COVID‐19 symptoms and the date of donation of the placenta and other clinical specimens (Figure 2A). These findings indicated that SARS‐CoV‐2 can be retained in the placentas of clinically recovered pregnant patients for nearly 3 months after the initial diagnosis.

### 3.3 Immune response in the placenta and maternal plasma

**FIGURE 3:**
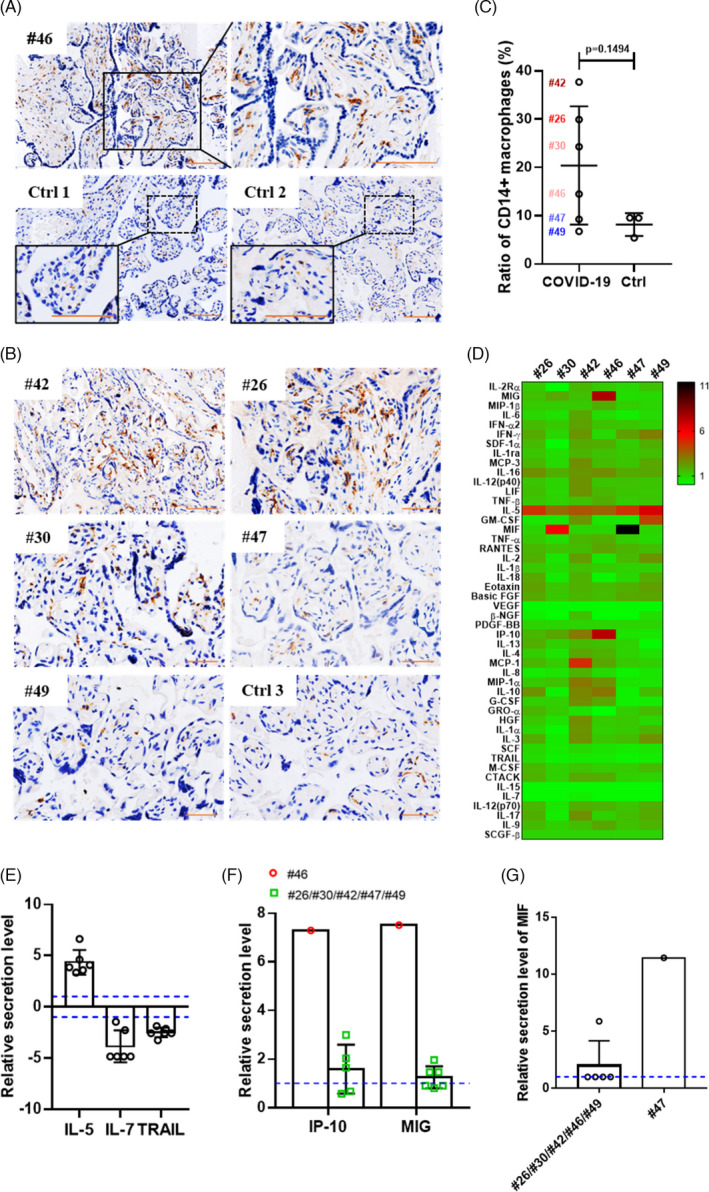
Inflammatory response in term placentas of pregnant women recovered from COVID‐19. A, Immunostaining for CD14+ macrophages in the term placenta of patient #46. Two placentas from uninfected pregnant women served as the negative controls (Ctrl 1 and Ctrl 2). Nuclei were stained blue with haematoxylin. Scale bars: 100 μm. B, Immunostaining for CD14+ macrophages in the placentas of five COVID‐19‐recovered pregnant patients (#42, #26, #30, #47 and #49). The placenta of an uninfected pregnant woman was used as the negative control sample (Ctrl 3). Scale bars: 50 μm. C, Ratio between CD14+ staining area and the area occupied by all cells in the placental villi of six recovered pregnant women (#26, #30, #42, #46, #47 and #49) and three uninfected pregnant women, indicating the extent of CD14+ macrophage infiltration in the placental villi. D, Heatmap showing the relative cytokine profiles of the indicated placentas evaluated using the Luminex assay compared with those of the placentas collected from four uninfected pregnant women. E, Differentially expressed cytokines in the placentas of COVID‐19‐recovered pregnant women compared with those of normal controls. F, The relative IP‐10 and MIG secretion levels increased in the placenta of patient #46 (red circles) compared with that in patients #26, #30, #42, #47 and #49 (green squares). G, The relative secretion level of MIF in the placenta of patient #47 was higher than that in the indicated placentas based on comparison with the level in normal controls. The blue dotted lines in E, F and G represent the baseline 1 or −1

After SARS‐CoV‐2 infection, the immune response in the patient is activated, and the infiltration of macrophages and lymphocytes into the infected locus is a common mechanism for virus clearance.^18^, ^35^ To clarify the nature of the immune response that might be activated by SARS‐CoV‐2 infection in the placenta, we performed immunohistochemical staining to study changes in the localization of CD14+ macrophages in the placentas that were positive (#26, #30, #42, #46 and #49) or negative (#47) for viral S protein, relative to the normal uninfected pregnant women. In the placenta. Among the macrophages localized within the placental villi are Hofbauer cells of foetal origin, which can be identified by CD14+ immunostaining. The infiltration of excess CD14+ macrophages in the placenta may indicate innate immune activation. As shown in Figure 3A and 3B, CD14+ macrophages were detected in all placentas, including those from patients #26, #30, #42, #46, #47 and #49 and from the three normal controls. Analysis of the ratio of CD14+ staining area to the total area occupied by the cells showed that compared with the placentas from patient #47 and the three normal controls, the placentas with residual viral nucleic acid or protein (#42, #26, #30 and #46) had elevated levels of CD14+ macrophages (Figure 3C), indicating a potential increase in innate immune activation in the placental villi after SARS‐CoV‐2 infection.^36^, ^37^ We also examined the distribution of CD3+ T lymphocytes in the placentas using the same method. However, CD3+ T lymphocytes were not detected in the placenta (Figure S2).

Immune activation in response to SARS‐CoV‐2 infection has been shown to induce cytokine secretion, which may be an important contributor to disease progression.^38^ The 48‐plex Luminex assay was performed to profile the cytokines and chemokines in the placentas of six patients (#26, #30, #42, #46, #47 and #49) and control placentas of uninfected pregnant women. At delivery, pronounced cytokine fluctuations were observed in all six placentas, and the synthesis of many cytokines was elevated to varying levels relative to that in normal controls (Figure 3D). All placentas showed elevated IL‐5 levels and decreased IL‐7 and TNF‐related apoptosis‐inducing ligand (TRAIL) levels (Figure 3E). Interestingly, the relative levels of IP‐10 and IFN‐γ (MIG) were higher in the placenta that tested positive for both viral nucleic acid and protein (#46) than in the placentas that tested positive only for the viral protein (#26, #30, #42, #47 and #49) (Figure 3F). The production of IP‐10 and MIG in local inflammatory lesions may attract Th1 cells, thereby leading to macrophage activation for virus clearance, which is consistent with the increased infiltration of CD14+ macrophages in infected placentas.^39^ The placenta of patient #47, which tested negative for both viral nucleic acid and protein, had a considerably higher level of migration inhibitory factor (MIF) than the five placentas with viral S protein (#26, #30, #42, #46 and #49; Figure 3G), which is indicative of intense SARS‐CoV‐2 clearance by macrophages.^40^ Taken together, the cytokine expression patterns in the placenta of pregnant women who had recovered from COVID‐19 suggested the activation of the antiviral immune response in the infected placentas.

**FIGURE 4:**
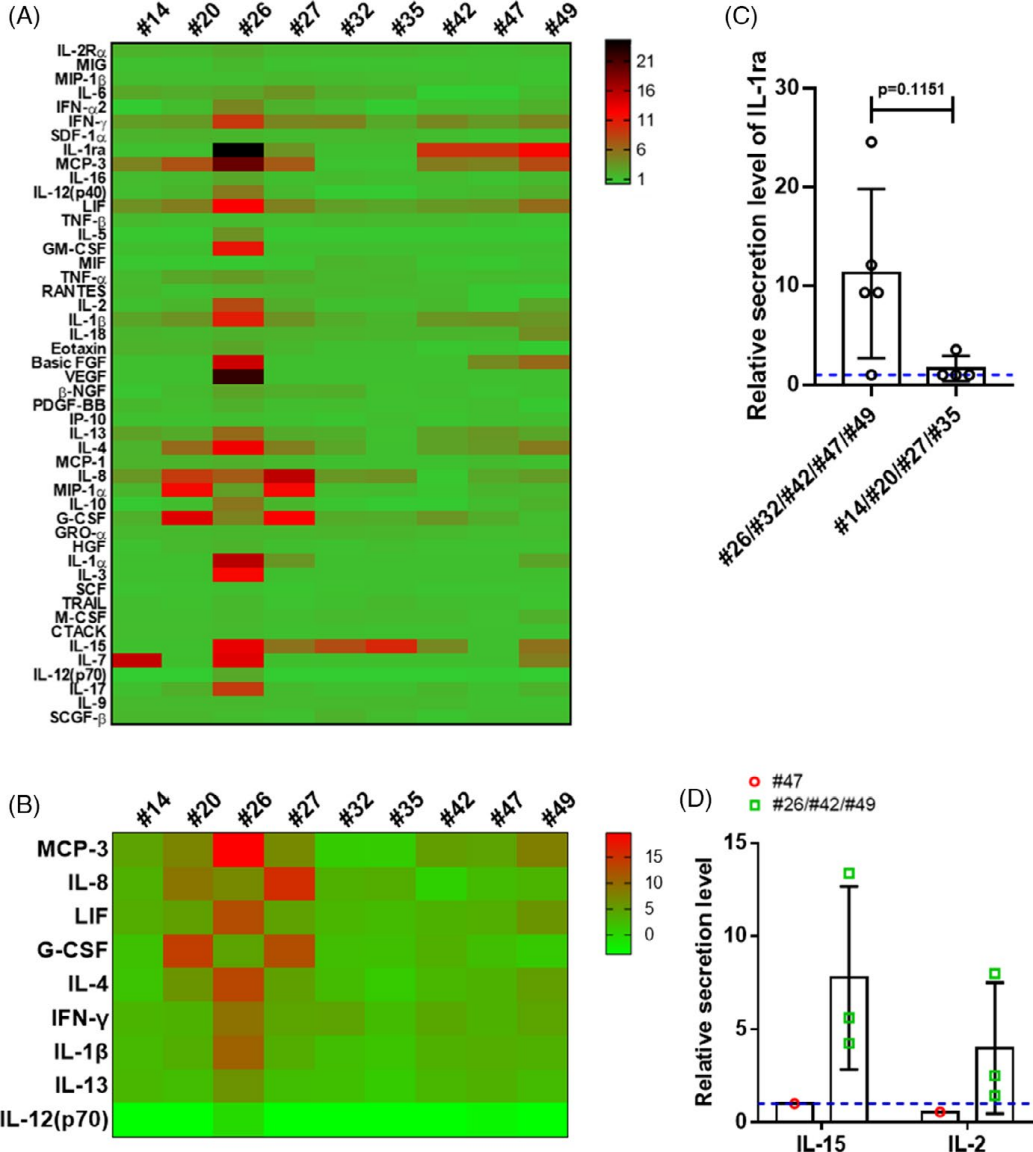
Cytokine profiles in maternal plasma at delivery. A, Heatmap showing the relative cytokine secretion profiles in the maternal plasma samples of nine COVID‐19‐recovered pregnant women (#14, #20, #26, #27, #32, #35, #42, #47 and #49), as detected using the Luminex assay, compared with the levels in the maternal plasma samples of three uninfected pregnant women. B, Heatmap showing differentially expressed cytokines in the plasma of nine recovered pregnant women at delivery compared with those in normal controls. C, The relative plasma IL‐1ra secretion levels in patients #26, #32, #42, #47 and #49, who showed normal chest CT results before delivery, was higher than those in patients #14, #20, #27 and #35, who showed convalescence in chest CT images. D, The relative plasma IL‐15 and IL‐2 secretion levels were elevated in patients #26, #42 and #49 (green squares), whose placentas tested positive for the viral S protein, compared with those in patient #47 (red circle). The blue dotted lines in C and D represent the baseline 1

To further explore the immune fluctuation in vivo, we profiled the cytokines in maternal plasma samples collected at delivery from nine recovered pregnant women (#14, #20, #26, #27, #32, #35, #42, #47 and #49) and three normal controls using the Luminex assay (Figure 4A). The relative secretion levels of MCP‐3, IL‐8, LIF, G‐CSF, IL‐4, IFN‐γ, IL‐1β and IL‐13 were higher, and those of IL‐12 (p70) were lower than those in normal controls (Figure 4B). Among these, MCP‐3, G‐CSF and IFN‐γ have been reported as being pro‐inflammatory mediators in ARDS caused by a ‘cytokine storm’.^41^, ^42^ The blockade of IL‐12, which is known to be a pro‐pathogenic cytokine, has been used for the treatment of autoimmune and autoinflammatory diseases.^43^ The reduction in IL‐12 levels in the plasma of recovered pregnant women may indicate the activation of an anti‐inflammatory response. Interestingly, the level of IL‐1 receptor antagonist (IL‐1ra), which can act as an antagonist to the pro‐inflammatory cytokine IL‐1 by binding to its receptor, was elevated in the maternal plasma samples collected from patients #26, #42, #47 and #49, who showed normal chest CT results at delivery; however, the IL‐1ra levels remained unchanged in the maternal plasma of patients #14, #20, #27 and #35, whose chest CT images indicated convalescence (Figure 4C and S3). Thus, the elevated IL‐1ra level may indicate an advanced inflammatory response, which was consistent with the CT imaging results.

Of the nine pregnant women who donated maternal plasma, four also donated their placentas (#26, #42, #47 and #49). Among them, the plasma IL‐2 and IL‐15 levels in patients #26, #42 and #49, who showed the presence of viral protein in the placenta, were elevated compared with those in normal controls (Figure 4D). Plasma IL‐2 signalling can stimulate the expansion of regulatory T cells,^44^ and IL‐15 is a known T‐cell growth factor,^44^ both IL‐2 and IL‐15 show overlapping activities in pathogen elimination.^44^ Collectively, we observed specific patterns of cytokine secretion in the plasma of pregnant women who had recovered from COVID‐19.

### 3.4 Clinical outcomes of pregnant women and newborns

To determine whether placental SARS‐CoV‐2 infection affected the pregnancy outcome and led to the intrauterine infection of the foetus, we further investigated the foetal and accessory abnormalities at delivery. Of all newborns, the monochorionic diamniotic twins weighed 2500 g and 2100 g, whereas the other full‐term infants had body weights ranging from 2810 g to 3900 g. All infants received 1‐minute and 5‐minute Apgar scores of 8 ‐ 10 (Table S3). Maternal infection‐associated abnormalities were not observed in the foetuses and appendages of any of the 11 pregnant women (Table S4).

To further explore the possibility of intrauterine infection of the foetus, we performed a COVID‐19 test on the newborns, and no viral nucleic acid was detected in the nasopharyngeal swab samples. However, seven newborns (#26, #27, #32, #35, #46, #47 and #49) tested positive for IgG but negative for IgM. None of the newborns showed typical symptoms of COVID‐19 pneumonia. In addition, the umbilical cords from five patients (#26, #27, #46, #47 and #49) and amniotic fluid from three patients (#26, #42 and #47) were collected for SARS‐CoV‐2 testing. All umbilical cord and two amniotic fluid samples (#26 and #47) tested positive for IgG, whereas none of the specimens tested positive for IgM (Table 2). These findings indicated the absence of obvious evidences of intrauterine infection.

**FIGURE 5:**
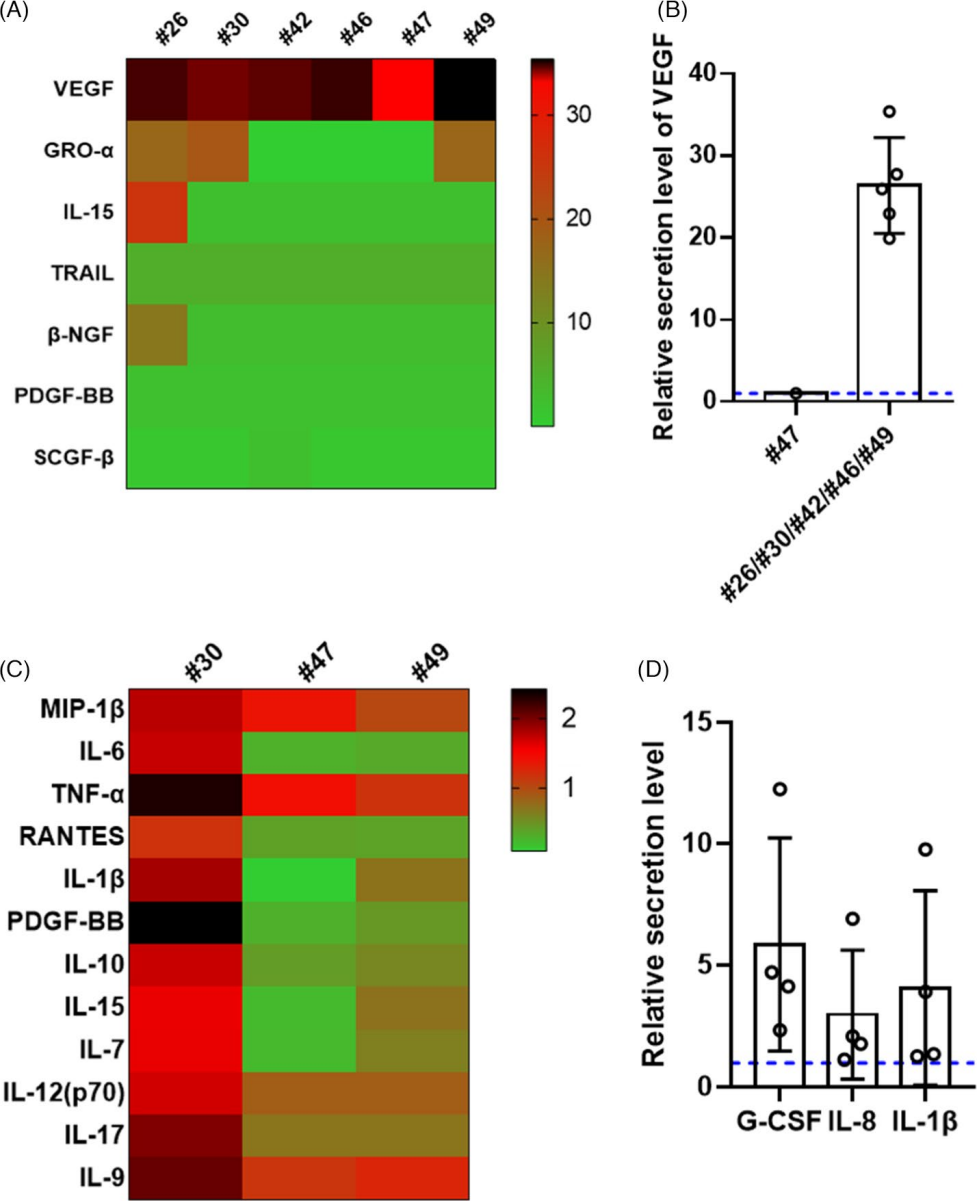
Cytokine profiles of umbilical cord, cord blood and amniotic fluid specimens of COVID‐19‐recovered pregnant women. A, Heatmap showing differentially expressed cytokines in the umbilical cords of six recovered pregnant women (#26, #30, #42, #46, #47 and #49) compared with those in the umbilical cords of two normal controls. B, The relative VEGF secretion levels in the umbilical cords of patients #26, #30, #42, #46 and #49 (placental villi tested positive for viral S protein) were elevated compared with that in the umbilical cord of patient #47. C, Heatmap showing multiple differentially expressed cytokines in the cord blood samples collected from three recovered pregnant women (#30, #47 and #49) compared with those in normal cord blood samples. D, The relative secretion levels of G‐CSF, IL‐8 and IL‐1β were elevated in the amniotic fluid of four COVID‐19‐recovered pregnant women (#26, #35, #47 and #49) compared with those in amniotic fluid specimens collected from uninfected women. The blue dotted lines in B and D represent the baseline 1

Next, we assessed whether maternal SARS‐CoV‐2 infection activated a neonatal immune response by analysing the cytokine secretion in neonatal umbilical cords, cord blood and amniotic fluid. Of the six umbilical cords tested (#26, #30, #42, #46, #47 and #49), the relative secretion levels of vascular endothelial growth factor (VEGF) were high in five samples, which were collected from the patients whose placental villi showed the presence of viral protein; the two normal controls were used for comparison (Figure 5A and 5B). In addition, the levels of GRO‐α, IL‐15, TRAIL, β‐NGF, PDGF‐BB and SCGF‐β secretion were generally lower (Figure 5A) than those in the controls. The relative cytokine profiles of the cord blood samples collected from three (#30, #47 and #49) newborns were considerably inconsistent with those of the two normal controls. The levels of multiple cytokines (MIP‐1β, IL‐6, TNF‐α, RANTES, IL‐1β, PDGF‐BB, IL‐10, IL‐15, IL‐7, IL‐12 (p70), IL‐17 and IL‐9) were higher in one neonatal cord blood sample (#30), whereas no significant changes were observed in the neonatal cord blood of the children of patients #47 and #49 (Figure 5C). The difference in cytokine secretion in the cord blood of patients #30, #47 and #49 was consistent with the difference in macrophage infiltration in the placental villi. More interestingly, the higher secretion levels of IL‐6, TNF‐α, IL‐10, IL‐12 (p70), IL‐15, IL‐17 and RANTES in the cord blood collected from patient #30 may indicate that T cell infiltration was involved in the immune response triggered by maternal infection.^45^, ^46^, ^47^, ^48^, ^49^, ^50^ Moreover, we tested the cytokine secretion in the amniotic fluid of four newborns (#26, #35, #47 and #49), and found that the relative secretion levels of G‐CSF, IL‐8 and IL‐1β were also higher than those in the three normal controls (Figure 5D). In conclusion, the fluctuation of cytokine levels in the neonatal umbilical cord, cord blood and amniotic fluid suggested that the neonatal innate immune response might have been activated in utero.

## 4 DISCUSSION

SARS‐CoV‐2 infection in the placenta and the placental pathology in COVID‐19‐positive mothers has been reported in previous studies.^51^ However, there is a lack of compelling evidence to confirm the vertical transmission of SARS‐CoV‐2. In this study, we used qPCR, immunostaining and the Luminex assay to study the persistence of SARS‐CoV‐2 nucleic acid and protein in the term placentas and the immune response in maternal plasma, placentas, umbilical cords, cord blood and amniotic fluid of women who had recovered from COVID‐19. We found that SARS‐CoV‐2 nucleic acid and protein are persistent in term placentas collected from clinically recovered pregnant patients, with specific localization observed in the villous syncytiotrophoblast. Even at 3 months of post‐diagnosis, SARS‐CoV‐2 nucleic acid and protein were detected in the placenta of some patients. On one hand, these results confirmed that the placenta is susceptible to SARS‐CoV‐2 infection, and once the virus infects the placenta, it persists for a considerable duration. Placental SARS‐CoV‐2 infection has also been reported in several studies.^52^, ^53^ However, vertical SARS‐CoV‐2 transmission has not been confirmed in most studies conducted thus far. On the other hand, previous studies have shown the presence of residual virus in the pneumocytes and infection‐induced pathological changes in the lungs of ready‐for‐discharge patients.^4^, ^54^ These results further indicate that the virus is far from being eliminated from the body of clinically recovered patients. Why does SARS‐CoV‐2 persist in the human body for such a long time? Jaenisch et al recently reported that SARS‐CoV‐2 RNA could integrate into the human genome and form a host‐virus chimeric (HVC) RNA,^55^ which may account for the prolonged detection of SARS‐CoV‐2 RNA; however, HVC is only present in rare cases, as reported in a study on the transcriptome of patients with COVID‐19.^56^ Therefore, the mechanisms underlying the persistence of the virus in the body of clinically recovered patients require further elucidation.

Several lines of evidence have shown that the adaptive immune response induced by SARS‐CoV‐2 infection can last for nearly 6 months.^57^, ^58^ Furthermore, recent studies have shown that in pregnant women, the anti‐SARS‐CoV‐2 antibody produced during maternal infection can be transferred to the foetus.^59^ In this study, we showed the persistent infiltration of CD14+ macrophages in infected placentas and continuous fluctuation of cytokine levels in the maternal and foetal specimens even at more than 3 months of post‐diagnosis. Even though no SARS‐CoV‐2‐associated foetal pathology was observed in the recovered pregnant women in this study, it remains to be determined whether the immune response‐related changes observed in the clinical samples collected from foetuses can contribute to adverse outcomes.

Collectively, our findings showed persistent SARS‐CoV‐2 infection and macrophage infiltration in the term placentas of pregnant women who had recovered from COVID‐19, suggesting a high probability of SARS‐CoV‐2 infection in human placentas.

## CONFLICT OF INTEREST

The authors declare there are no competing interests and all authors consent to publish the data.

## AUTHORS’ CONTRIBUTIONS

H. M. W., S. L., D. D., Y. Z. and Z. X. conceived the project. H. M. W. and Z. X. supervised the project. S. L., Y. Z., D. D., X. L., J. W., R. W., D. L., D. W., M. H., B. H., R. L., J. P., H. Y., H. Y., Y. Z., W. Z., X. W., Z. H. and K. L. collected clinical specimens. H. W., Y. W. and M. G. conducted experiments and data analysis. H. W. and Z. X. wrote the manuscript.

## Supporting information

Supplementary Material

## Data Availability

The data that support the findings of this study are available from the corresponding author upon reasonable request.
