# Supplementary material for: Molecular evidence suggesting the persistence of residual SARS‐CoV‐2 and immune responses in the placentas of pregnant patients recovered from COVID‐19
Source: Cell Prolif. 2021 Jul 22;54(9):e13091. doi: 10.1111/cpr.13091 (PMC8420381; doi:10.1111/cpr.13091)
Supplement: Supplementary file 1 — Supplementary Material [file CPR-54-e13091-s001.docx]

**Supporting Information**

**Molecular evidence suggesting the persistence of residual SARS-CoV-2 and immune responses in the placentas of pregnant patients recovered from COVID-19**

Hao Wu^1,5,6,7,†^, Shujie Liao^2,†,‡^, Yiming Wang^1,5,6,7,†^, Ming Guo^3,†^, Xingguang Lin^2^, Jianli Wu^2^, Renjie Wang^2^, Dan Lv^2^, Di Wu^2^, Mengzhou He^2^, Bai Hu^2^, Rui Long^2^, Jing Peng^4^, Hui Yang^4^, Heng Yin^4^, Xin Wang^3^, Zhixiang Huang^3^, Ke Lan^3^, Yanbin Zhou^8^, Wei Zhang^9^, Zhenyu Xiao^1,5,7,‡^, Yun Zhao^4,‡^, Dongrui Deng^2,‡^, Hongmei Wang^1,5,6,7,‡^

1. State Key Laboratory of Stem Cell and Reproductive Biology, Institute of Zoology, Chinese Academy of Sciences, 100101 Beijing, China.

2. Department of Gynecology and Obstetrics, Tongji Hospital, Tongji Medical College, Huazhong University of Science and Technology, 430030 Wuhan, Hubei, China.

3. State Key Laboratory of Virology, Modern Virology Research Center, College of Life Sciences, Wuhan University, 430072 Wuhan, Hubei, China.

4. Department of Obstetrics, Maternal and Child Health Hospital of Hubei Province, Tongji Medical College, Huazhong University of Science and Technology, 430070 Wuhan, Hubei, China.

5. Institute for Stem Cell and Regeneration, Chinese Academy of Sciences, 100101 Beijing, China.

6. University of Chinese Academy of Sciences, 100049 Beijing, China.

7. Beijing Institute for Stem Cell and Regenerative Medicine, Beijing 100101, China

8. Department of Obstetrics and Gynecology, People’s Hospital of Huangmei Country, Huanggang City, Hubei, China.

9. Zhongnan Hospital, Wuhan University, 430071 Wuhan, Hubei, China.

† These authors contributed equally to this article.

‡ Co-corresponding authors.

**Correspondence:**

Hongmei Wang and Zhenyu Xiao, State Key Laboratory of Stem Cell and Reproductive Biology, Institute of Zoology, Chinese Academy of Sciences 100101 Beijing, China.

Emails: [wanghm@ioz.ac.cn](mailto:wanghm@ioz.ac.cn); [xiaozy@ioz.ac.cn](mailto:xiaozy@ioz.ac.cn)

Shujie Liao and Dongrui Deng, Department of Gynecology and Obstetrics, Tongji Hospital, Tongji Medical College, Huazhong University of Science and Technology, 430030 Wuhan, Hubei, China.

Emails: [sjliao@tjh.tjmu.edu.cn](mailto:sjliao@tjh.tjmu.edu.cn); [dr.deng@tjh.tjmu.edu.cn](mailto:dr.deng@tjh.tjmu.edu.cn)

Yun Zhao, Department of Obstetrics, Maternal and Child Health Hospital of Hubei Province, Tongji Medical College, Huazhong University of Science and Technology, 430070 Wuhan, Hubei, China.

Email: [zhao020060@163.com](mailto:zhao020060@163.com)


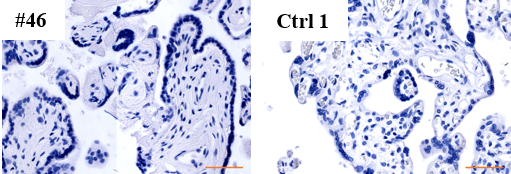


**Figure S1 Immunostaining of paraffinized sections of tissues collected from patients or uninfected pregnant woman (controls) after elimination of the primary antibody.** Scale bars: 50 μm.


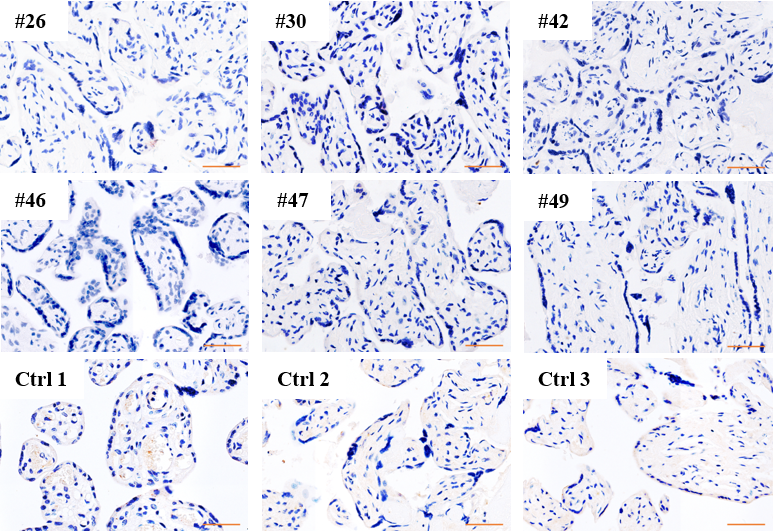


**Figure S2 Immunohistochemical staining for CD3+ T lymphocytes in the indicated term placental villi.** Staining for CD3, a T lymphocyte marker, revealed no T lymphocyte infiltration in the intervillous and intravillous spaces. Scale bars: 50 μm.

**
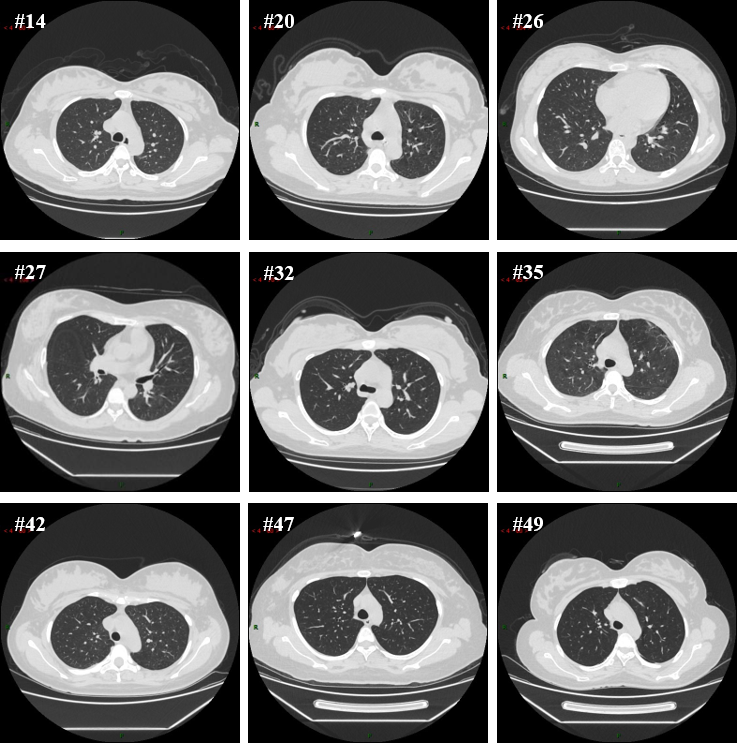
**

**Figure S3 Chest CT images showing the lung structure of patients before delivery.**

| **Table S1. Treatments for the pregnant women diagnosed with COVID-19.** | | | | | | | | | | | |
| --- | --- | --- | --- | --- | --- | --- | --- | --- | --- | --- | --- |
|  | **#14** | **#20** | **#26** | **#27** | **#30** | **#32** | **#35** | **#42** | **#46** | **#47** | **#49** |
| Admission to intensive care unit | No | No | No | No | No | No | No | No | No | No | No |
| Oxygen therapy | No | No | No | Yes | No | No | Yes | No | No | No | No |
| Antibiotic therapy | Yes | Yes | No | Yes | No | No | Yes | Yes | Yes | Yes | No |
| Cephalosporin | Yes | Yes | No | Yes | No | No | Yes | Yes | Yes | Yes | No |
| Ornidazole | Yes | Yes | No | No | No | No | No | No | No | Yes | No |
| Antiviral therapy | No | No | No | No | No | No | No | No | Omeprazole | No | No |
| Systemic glucocorticoids | No | No | No | No | No | No | No | No | No | No | No |
| Mechanical ventilation | No | No | No | Yes | No | No | Yes | No | No | No | No |
| Invasive | No | No | No | No | No | No | No | No | No | No | No |
| Noninvasive | No | No | No | Yes | No | No | Yes | No | No | No | No |
| Use of intravenous immune globulin | No | No | No | No | No | No | No | No | No | No | No |

| **Table S2. Prenatal testing results.** | | | | | | | | | | | | | |
| --- | --- | --- | --- | --- | --- | --- | --- | --- | --- | --- | --- | --- | --- |
|  | | **#14** | **#20** | **#26** | **#27** | **#30** | **#32** | **#35** | **#42** | **#46** | **#47** | **#49** | **Reference range** |
| **Blood routine examinations** | | | | | | | | | | | | | |
| White-cell count (×10^9^/L) | | 9.97 | 11.66 | 10.44 | 13.03 | 7.84 | 9.73 | 11.83 | 9.82 | 9.7 | 12.17 | 5.05 | 6-10 ╨ |
| Lymphocyte count (×10^9^/L) | | 0.98 | 0.99 | 1.75 | 1.8 | 1.62 | 1.06 | 1.52 | 1.29 | 1.95 | 1.22 | 1.57 | 1.1-3.2 ╨ |
| Hemoglobin (g/L) | | 96 | 121 | 108 | 120 | 106 | 105 | 148 | 136 | 122 | 126 | 115 | 100-130 ╨ |
| Platelet count (×10^9^/L) | | 270 | 173 | 268 | 210 | 208 | 196 | 172 | 270 | 131 | 185 | 249 | 125-350 ╨ |
| **Liver function examinations** | | | | | | | | | | | | | |
| ALT (U/L) | | 17.5 | 6.8 | 5.6 | 29.7 | N/A | 9.4 | 10.0 | 18.5 | 8.8 | 13.3 | 5.3 | 7-40 ╨ |
| AST (U/L) | | 22.1 | 18.3 | 14.6 | 29.8 | N/A | 20.7 | 20.4 | 23.0 | 14.5 | 24.4 | 15.0 | 13-35 ╨ |
| Albumin (g/L) | | 35.8 | 33.1 | 36.4 | 40.1 | N/A | 36.4 | 35.2 | 36.2 | 37.8 | 37.0 | 39.9 | 40-55 ╨ |
| TB (μmol/L) | | 12.8 | 9.1 | 5.3 | 5.3 | N/A | 6.6 | 7.9 | 6.7 | 4.8 | 4.7 | 19.1 | 3.4-20.5 ╨ |
| GGT (U/L) | | 13.0 | 7.7 | 8.5 | 44.9 | N/A | 13.6 | 15.1 | 12.0 | 7.0 | 14.7 | 6.0 | 7-45 ╨ |
| LDH (U/L) | | 152.8 | 239.5 | 184 | 184.5 | N/A | 196.5 | 248.4 | N/A | 172 | 187.5 | 189 | 120-250 ╨ |
| **Renal function examinations** | | | | | | | | | | | | | |
| BUN (mg/dL) | | 2.98 | 5.19 | 2.64 | 3.45 | N/A | 4.43 | 3.52 | 1.90 | 3.8 | 4.99 | 4 | 2.6-7.5 ╨ |
| Creatinine (μmol/L) | | 38.7 | 41 | 55.5 | 48.1 | N/A | 55.4 | 50.8 | 49.0 | 45.0 | 49.9 | 49.0 | 41-73 ╨ |
| Uric acid (μmol/L) | | 2.98 | 545.1 | 251.4 | 330.1 | N/A | 377 | 278.3 | 211 | 319 | 508.5 | 238 | 155-357 ╨ |
| **Blood coagulation examinations** | | | | | | | | | | | | | |
| Prothrombin time (s) | | 10.7 | 10.7 | 10.6 | 11.2 | N/A | 10.4 | 11.5 | 10.1 | 10.1 | 10.2 | 10.7 | 11.5-14.3 ╨ |
| APTT (s) | | 26.4 | 26.2 | 26.0 | 25.1 | N/A | 26.1 | 32.5 | 26.7 | 26.8 | 23.5 | 24.5 | 28-40 ╨ |
| D-dimer (mg/L) | | 1.58 | 2.61 | 1.06 | 2.16 | N/A | 1.55 | 1.94 | 5.71 | 5.34 | 1.53 | 2.3 | ≤ 3.14 |
| **Cardiac function examinations** | | | | | | | | | | | | | |
| CK (U/L) | | 70.02 | 67.44 | 33.32 | 39.26 | N/A | 59.16 | N/A | N/A | 22 | 59.03 | 27 | 40-200 ╨ |
| CK-MB (U/L) | | 29.12 | 26.67 | 14.47 | 8.93 | N/A | 12.09 | N/A | N/A | 10.8 | 10.05 | 10.2 | <10 ╬ |
| CRP (mg/L) | | 65.18 | 30.23 | 1.46 | 87.1 | 95.5 | N/A | 28.9 | 3.95 | 5.13 | 155.75 | 0.56 | 0-5 ╨ |
| **Prenatal SARS-CoV-2 and chest CT examinations** | | | | | | | | | | | | | |
| Nucleic acid of SARS-CoV-2 in throat swab | | Negative | Negative | Negative | Negative | Negative | Negative | Negative | Negative | Negative | Negative | Negative |  |
| Anti-SARS-CoV-2 antibodies in serum | | IgG+/IgM- | IgG+/IgM- | IgG+/IgM- | IgG+/IgM- | IgG+/IgM- | IgG+/IgM- | IgG+/IgM- | IgG+/IgM- | IgG+/IgM- | IgG+/IgM- | IgG+/IgM- |  |
| Chest CT variation trend | | Dissipate | Dissipate | Normal | Dissipate | Dissipate | Normal | Dissipate | Normal | Normal | Normal | Normal |  |
| **N/A** | **Not applicable.** | | | | | | | | | | | | |
| **ALT** | **Alanine aminotransferase.** | | | | | | | | | | | | |
| **AST** | **Aspartate aminotransferase.** | | | | | | | | | | | | |
| **TB** | **Total bilirubin GGT.** | | | | | | | | | | | | |
| **GGT** | **Gamma-glutamyl transferase.** | | | | | | | | | | | | |
| **LDH** | **Lactate dehydrogenase.** | | | | | | | | | | | | |
| **BUN** | **Blood urea nitrogen.** | | | | | | | | | | | | |
| **APTT** | **Activated partial thromboplastin time.** | | | | | | | | | | | | |
| **CK** | **Creatine kinase.** | | | | | | | | | | | | |
| **CK-MB** | **Creatine kinase MB.** | | | | | | | | | | | | |
| **CRP** | **C-reactive protein.** | | | | | | | | | | | | |
| **╨** | **Refer to Obstetrics and Gynecology (9th edition) (Beijing: People' s Medical Publishing House, 2018).** | | | | | | | | | | | | |
| **╬** | **Refer to Diagnostics (9th edition) (Beijing: People' s Medical Publishing House, 2018).** | | | | | | | | | | | | |

| **Table S3. Perinatal and neonatal outcomes.** | | | | | | | | | | | | |
| --- | --- | --- | --- | --- | --- | --- | --- | --- | --- | --- | --- | --- |
|  | | **#14** | **#20** | **#26** | **#27** | **#30** | **#32** | **#35** | **#42** | **#46** | **#47** | **#49** |
| Pregnancy outcomes | | CS | CS | Eutocia | CS | CS | Eutocia | Eutocia | Odinopoeia | Eutocia | CS | Eutocia |
| Preterm delivery ┤ | | No | Yes | No | No | No | No | No | No | No | No | No |
| Gestational age at delivery (W) | | 38^+5^W | 36^+4^W | 39^+0^W | 38^+5^W | 38^+1^W | 39^+6^W | 39^+2^W | 38^+5^W | 38^+4^W | 39^+0^W | 39^+4^W |
| Birthweight (g) | | 3570 | 2500/2100 | 3670 | 3200 | 3100 | 3450 | 3900 | N/A | 2945 | 2940 | 2810 |
| Apgar score (1 min) ┼ | | 8 | 9 | 10 | 10 | 8 | 10 | 10 | N/A | 10 | 10 | 10 |
| Apgar score (5 min) ┼ | | 10 | 10 | 10 | 10 | 9 | 10 | 10 | N/A | 10 | 10 | 10 |
| IVF | | No | No | No | No | No | No | No | Yes | No | No | No |
| Twins | | No | Yes | No | No | No | No | No | Yes | No | No | No |
| **N/A** | **Not applicable.** | | | | | | | | | | | |
| **CS** | **Caesarean section.** | | | | | | | | | | | |
| **W** | **Week.** | | | | | | | | | | | |
| **g** | **Gram.** | | | | | | | | | | | |
| **IVF** | **In-vitro fertilization.** | | | | | | | | | | | |
| **┤** | **< 37W.** | | | | | | | | | | | |
| **┼** | **Refer to Pediatrics (9th edition) (Beijing: People' s Medical Publishing House, 2018).** | | | | | | | | | | | |

| **Table S4. Abnormal fetus and appendages.** | | | | | | | | | | | |
| --- | --- | --- | --- | --- | --- | --- | --- | --- | --- | --- | --- |
|  | **#14** | **#20** | **#26** | **#27** | **#30** | **#32** | **#35** | **#42** | **#46** | **#47** | **#49** |
| Threatened abortion | No | No | No | No | Yes | No | No | No | No | No | No |
| Placenta previa | No | No | No | No | No | No | No | No | No | Yes | No |
| PROM | No | No | No | No | No | No | No | No | No | No | No |
| Cord entanglement | No | No | No | No | Yes | No | No | No | No | No | No |
| Polyhydramnios | No | No | No | No | No | No | No | No | No | No | No |
| Oligoamnios | No | No | No | No | Yes | No | No | No | No | No | No |
| Fetal distress | No | No | No | No | No | No | No | No | No | No | No |
| Meconium-stained amniotic fluid | No | No | Ⅲ | No | No | No | No | No | No | No | No |
| Postpartum hemorrhage | No | No | No | No | No | No | No | No | No | No | No |
| Others | No | No | Accessory cyst | No | No | No | No | Malformation | No | No | No |
| **PROM Premature rupture of membranes.** | | | | | | | | | | | |
